# Markers Associated with Sex Differences in Methamphetamine-Induced Striatal Dopamine Neurotoxicity

**DOI:** 10.2174/157015911795017399

**Published:** 2011-03

**Authors:** D. E Dluzen, J. L McDermott, M Bourque, T Di Paolo, A. S Darvesh, A. B Buletko, N.J Laping

**Affiliations:** a Department of Anatomy and Neurobiology, NEOUCOM, Rootstown, OH 44272; b Molecular Endocrinology and Genomic Research Center and Department of Pharmacy, Laval University, Quebec City, QC, G1K 7P4; c Departments of Pharmaceutical Sciences and Psychiatry, NEOUCOM, Rootstown, OH 44272; d Endo Pharmaceuticals, Chadd’s Ford, PA 19317, USA

**Keywords:** Body Weight, Body Temperature, GFAP, Bcl-2 PAI-1, IGF-1R, Dopamine Transporter.

## Abstract

Three different approaches were employed to assess various markers associated with sex differences in responses to methamphetamine (MA). Bioassay measures reveal that MA treatment results in significantly greater reductions in body weight and increases in body temperature in male mice. Protein and mRNA determinations show significant increases in Bcl-2 and PAI-1 in male mice, while females show significant increases in GFAP and decreases in IGF-1R following treatment with MA. In mice with a heterozygous mutation of their dopamine transporter (+/- DAT), only female mice show significant differences in dopamine transporter binding and mRNA and associated reductions in striatal dopamine content along with increases in MA-evoked striatal dopamine output. The identification of these sex-dependent differences in markers provides a foundation for more exhaustive evaluation of their impact upon, and treatment of, disorders/neurotoxicity of the nigrostriatal dopaminergic system and the bases for the differences that exist between females and males.

## INTRODUCTION

Methamphetamine (MA) produces an acute increase in striatal dopamine (DA) output [1] and a long-lasting depletion of striatal DA concentration, which is greater in male mice [2, 3]. Analogous gender differences exist in the clinical literature. Women show lower amphetamine-evoked DA responses [4] and MA-induced toxicity is less pronounced in women [5]. Accordingly, significant sex differences exist with regard to uses of, and responses to, MA [6]. In this report we review our data on various parameters and signals as related with sex differences in MA-induced neurotoxicity upon striatal DA.

### Part 1: Bioassays 

**Fig. (1):**
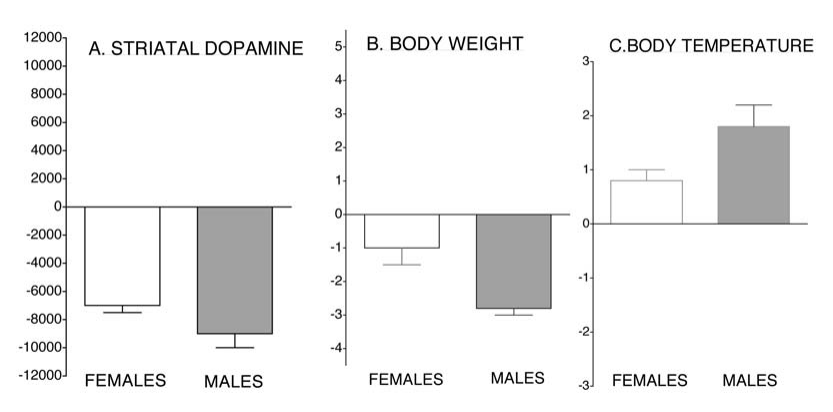
Relationships (Mean and SEM) among methamphetamine (MA – 40 mg/kg)-induced striatal dopamine depletions (**A** - pg/mg), body weights (**B** - gm) and body temperatures (**C** - °C) in female and male mice.

There are two biological measures that reveal sex differences as associated with MA-induced neurotoxicity – body weights and temperature. When comparing changes in body weight from the immediate pre-MA treatment level with that at 3-5 days post-MA treatment, there is a statistically significant, approximately 1.0 gm greater reduction in body weights of male mice (Fig. **1B**) as associated with increased striatal DA depletions (Fig. **1A**).

There exists a fairly extensive literature base on the variable of temperature and effects of MA [7]. We reported that estrogen treatment, which significantly diminishes MA- induced neurotoxicity in female, but not male, mice, significantly reduces body temperature only in female mice [8]. In response to MA (40 mg/kg), both females and males show increases in body temperature, however, this increase was significantly greater in males (Fig. **1C**). Taken together these two bioassays reveal that in male mice, where an increased amount of striatal DA depletion is present to MA, there are corresponding increases in body weight reductions and increases in body temperature.

### Part 2: Protein and mRNA Levels

#### Glial Fibrillary Acidic Protein (GFAP)

**Fig. (2):**
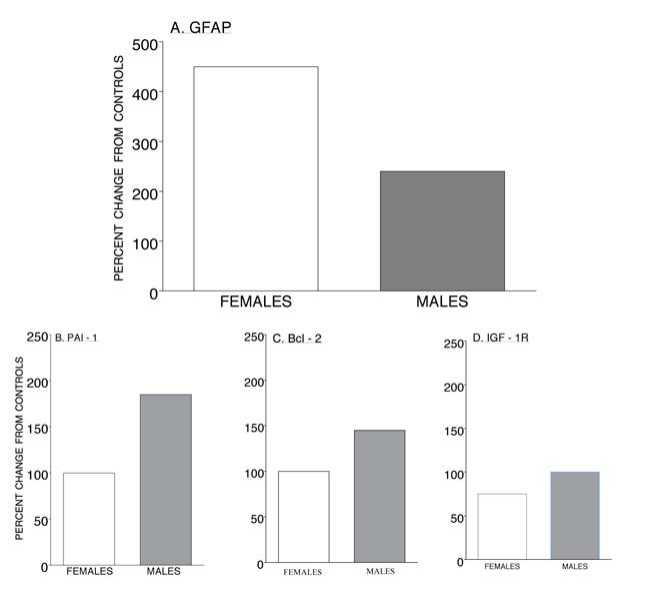
Summary of changes (percents) in markers as determined from striatal tissue of methamphetamine-treated female and male mice. A. GFAP mRNA in males is approximately two-fold greater than females, but following methamphetamine treatment, GFAP levels are approximately 50% that of females. Levels for both PAI-1 (B) and Bcl-2 (C) were significantly increased in males. Levels of IGF-1R (D) were significant decreased in females. Data derived from references # [2, 7, 16].

There exist some particularly intriguing changes in reactive astrocytes as measured by GFAP between MA-treated female and male mice. Although basal levels of GFAP are greater in males [7], striatal GFAP mRNA was approximately two-fold greater in females (p = 0.06) at 7-days after MA (4 X 20 mg/kg @ 2 h intervals) (Fig. **2A**) [2]. In these same mice, striatal DA, while severely depleted to approximately 20% of control values, remained significantly greater in females. These results were somewhat unexpected, but very similar to that reported for female and male mice treated with d-MDMA. With this treatment, male mice showed a more severe depletion of striatal DA (~ 10% of controls) accompanied by an approximately 2.7-fold increase in GFAP protein, while females showed a less severe DA depletion (~ 30% of controls) accompanied by an approximately 5.8-fold increase in GFAP protein [7]. When this information on the sex differences in striatal DA and GFAP is collated a significant relative sex differential in GFAP is present in response to MA; that is, the approximately two-fold *greater* levels of GFAP that are observed in males under basal conditions become approximately two-fold *lower* in males following MA treatment. The enhanced GFAP response that we captured in females at this time point post-MA may reveal a manner of restorative activity in astrocytes to prevent further decline in such functions as blood brain barrier disruption or demyelination of the nervous system.

#### Plasminogen Activator Inhibitor-1 (PAI-1)

At 7 days post-MA, when striatal DA concentrations of female mice were significantly greater than males, an approximately 1.85-fold and statistically significant increase in PAI-1 was obtained in male mice, with no change in females (Fig. **2B**) [2]. In general, basal levels of PAI-1 are greater in males [9] and men seem more responsive or sensitive to PAI-1 relationships [10]. Repeated MA administration produced significant, dose-dependent increases in tissue plasminogen activator mRNA in both the nucleus accumbens and striatum of mice [11]. Our findings appear relatively congruent with that reported by others in that PAI-1 is more prevalent in males and, in turn, males seem more sensitive with regard to PAI-1 responses of stress or insult. The increased PAI-1 response in males may represent a PAI-1 activation by TGF-ß protein in the context of MA-evoked glutamate to result in the enhanced neurotoxicity and/or reflect an ongoing inflammatory response as associated with the increased neurotoxicity of males.

#### Bcl-2

When sex differences were evaluated for Bcl-2 in response to MA, we found that striatal levels were significantly increased in male, but not female, mice at 7 days post-treatment with 40 mg/kg of MA (Fig. **2C**). Striatal Bcl-2/BAX ratios were significantly greater in wild type control (parkin) females, which appeared to be due mostly to lower BAX levels in females [12]. The increase in Bcl-2 we observed in male mice at 7 days post-MA differs from decreases typically seen at much earlier time points post-MA. Male mice treated with MA (4 X 10 mg/kg @ 2h intervals) showed a significant down regulation in striatal Bcl-2 expression when sampled at 72 h post-MA [13]; and in immortalized striatal cell cultures, Bcl-2 was significantly decreased at 24 h post-treatment with 2 mM MA [14]. Since increased expression of Bcl-2 decreased the temporal- and dose-dependent loss of cell viability from immortalized cells exposed to MA [15], it was somewhat surprising to see these levels increased in males, where augmented neurotoxicity is present. The timing of Bcl-2 determinations might represent an important factor with regard to levels obtained and the elevated levels of Bcl-2, along with PAI-1, may indicate a need for a stronger recovery or compensatory response to MA in the male.

#### Insulin-Like Growth Factor-1 Receptor (IGF-1R).

Administration of MA at either 20 or 40 mg/kg significantly decreased striatal IGF-1R levels in female, but not male mice, as determined at 7-days post-MA treatment (Fig. **2D**). The enhanced sensitivity in females may be of particular significance where a coactivation of estrogen and IGF-1 receptors seem to be involved with neuroprotection. Moreover, the capacity for IGF-1 to synergize with estrogen for activation of the mitogen-activated protein kinase (MAPK) and phosphoinositide 3-kinase (PI3K)/Akt pathways represent critical signal transduction casacades for neuroprotective actions [16]. 

#### pAkt and GSK 3 ß

These two markers showed an overall, predominant MA-effect. In both females and males these markers were decreased following MA treatment, with the degree of decrease being slightly greater in males. In the case of pAkt, this reduction achieved statistical significance (30% decrease). Such a reduction in pAkt might reflect a diminished activation of survival pathways due to the increased capacity for MA to decrease striatal DA and GFAP astrocyte expression in males. A number of markers indicated neither a sex difference nor effect of MA (Activin-like kinase 5, Akt, BAD, ERK 1 and 2, pERK 1 and 2, Fibronectin, pGSK 3ß, TGFß and TGFß R2). 

### Part 3: +/- Dopamine Transporter KO Model

**Fig. (3):**
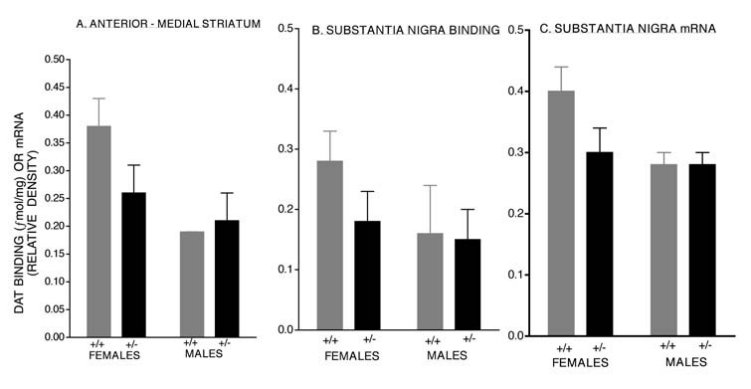
Dopamine Transporter (DAT) specific binding (Mean and SEM in fmol/mg of tissue) within the medial region of the anterior striatum (**A**), substantia nigra (**B**) and DAT mRNA (relative optical density) within the substantia nigra (**C**) of +/+ DAT control and heterozygous mutant (+/- DAT) female and male mice. Levels from +/+ DAT females were significantly greater than all other conditions tested and only +/- DAT females showed significant decreases in DAT binding and mRNA. Data derived from reference # [20].

**Fig. (4):**
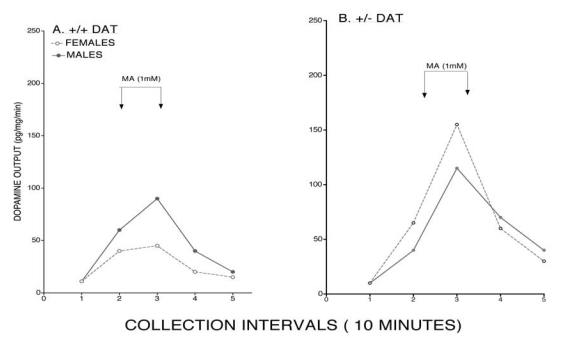
Methamphetamine-evoked dopamine output (Mean - pg/mg/min) of +/+ DAT control and heterozygous mutant (+/- DAT) female and male mice. While methamphetamine-evoked dopamine of +/+ mice was significantly greater in males, this trend was reversed in the +/- DAT mice. Data derived from reference # [21].

Given that a primary site for MA’s action is upon the dopamine transporter (DAT) and females show greater DAT function [17] and densities of DATs as reported in both laboratory [18] and clinical [19] studies, we evaluated sex differences in dopaminergic function in mice with a DAT mutation. A consistent finding resulting from comparisons of heterozygous mutant DAT (+/- DAT) mice is that this mutation does not exert equivalent effects upon the dopaminergic system of females and males. Significant reductions are consistently seen in +/- DAT female, but not male, mice for striatal DAT binding and substantia nigra binding and mRNA (Fig. **3**) [20]. As a result, in +/- DAT female, but not male, mice striatal DA concentrations and MA-evoked dopamine responses are altered [21]. While MA-evoked DA responses are greater in wild type males, this profile was reversed when tested in +/- DAT mice (Fig. **4**). It seems as though males are capable of generating compensatory responses which enables them to maintain their dopaminergic responses in the presence of a DAT allele deletion. These findings have important implications as changes in the DAT that can occur with age or in conditions like MA abuse or Parkinson’s disease may have decidedly different consequences for females and males.
